# The Effect of a Customized Nutrient-Profiling Approach on the Glycated Hemoglobin Levels of Patients With Type 2 Diabetes: Quasi-Experimental Study

**DOI:** 10.2196/15497

**Published:** 2020-05-19

**Authors:** Mayda Alrige, Riad Alharbey, Samir Chatterjee

**Affiliations:** 1 King Abdulaziz University Jeddah Saudi Arabia; 2 University of Jeddah Jeddah Saudi Arabia; 3 Claremont Graduate University Claremont, CA United States

**Keywords:** mHealth, consumer health informatics, diet therapy, nutrient profiling, Hb A1c

## Abstract

**Background:**

Presently, dietary management approaches are mostly oriented toward using calorie-counting and diet-tracking tools that draw our attention away from the nutritional value of our food. To improve individuals’ dietary behavior, primarily that of people with type 2 diabetes, a simple technique is needed to increase their understanding of the nutritional content of their food.

**Objective:**

This study aimed to design, develop, and evaluate a customized nutrient-profiling tool called *EasyNutrition*. *EasyNutrition* was built to introduce the new concept of nutrient profiling by applying the Intelligent Nutrition Engine, an algorithm that we developed for ranking different food recipes based on their nutritional value. This study also aimed to investigate the efficacy of *EasyNutrition* in lowering glycated hemoglobin (HbA_1c_) levels and improving dietary habits among people with type 2 diabetes.

**Methods:**

We evaluated the utility of *EasyNutrition* using design science research in three sequential stages. This paper has elaborated on the third stage to investigate the efficacy of *EasyNutrition* in managing type 2 diabetes. A quasi-experimental study was conducted in a diabetes treatment center (n=28). The intervention group utilized *EasyNutrition* over 3 months, whereas participants in the control group utilized the standard of care provided by the center. Dietary habits and HbA_1c_ levels were measured to capture any change before and after experimenting with *EasyNutrition*.

**Results:**

The intervention group (n=9) exhibited a statistically significant change between the pre- and postexposure results of their HbA_1c_ (*t*_9_=2.427; *P*=.04). Their HbA_1c_ dropped from 8.13 to 6.72. This provided preliminary evidence of the efficacy of using a customized nutrient-profiling app in reducing HbA_1c_ for people with type 2 diabetes.

**Conclusions:**

This study adds to the evidence base that a nutrient-profiling strategy may be a modern adjunct to diabetes dietary management. In conjunction with reliable dietary education provided by a registered dietician, *EasyNutrition* may have some beneficial effects to improve the dietary habits of people with type 2 diabetes.

## Introduction

### Problem Domain

Keeping up with a healthy, well-balanced diet is by no means easy. This is primarily challenging for patients with type 2 diabetes because it is related to maintaining a certain metabolic goal. The traditional dietary methods to manage diabetes include carbohydrate intake monitoring and *my plate*, among many others. Despite their effectiveness when followed strictly by patients, these methods are very cumbersome to deal with on a daily basis. The methods are based on specifying the quantity of certain nutrients. For example, following the *my plate* method, users must adhere to 25% protein, 25% grain and starchy foods, and 50% nonstarchy vegetables. For certain recipes, it is not practical to calculate those percentages. Dealing with quantities to manage diets is very tedious and time consuming.

Another problem with the current traditional dietary management methods is the focus on single nutrients instead of the overall nutritional value. Carbohydrate-restricted, fat-restricted diets are examples of these kinds of diets. The American Diabetes Association (ADA) released a new statement in 2013 summarizing the nutrition therapy recommendations for people with type 2 diabetes. As people eat food and no single nutrient such as carbohydrates, protein, and fat, this new statement designates a new section on eating patterns or plans. Patients with diabetes can still enjoy the food they like while, at the same time, keeping their diabetes under control. In addition, educating people about nutrition has to be aimed at modifying the factors that influence their dietary behavior rather than being aimed at increasing their knowledge about nutrition. Bader et al [1] found, through their comprehensive literature review, that knowledge-based nutrition education programs alone do not result in dietary behavior change. For instance, stating the recommended carbohydrate intake might increase one’s knowledge about nutrition but not influence his or her dietary behavior to consume fewer carbohydrates. When this piece of information is applied in recipes and presented in a practical way, the chances are that patients would apply these tips and keep using them. This is the rationale behind behavioral nutrition, which is to change the target of nutrition education from the goal of increasing knowledge to that of modifying factors that influence dietary behaviors.

In addition, one more issue concerning the current dietary management tools is identified by the ADA in their new position statement on nutrition therapy recommendations. The ADA states that there is no single eating pattern that is best for everyone [2]. In their 2013 report, individualization is highlighted as a key element, as different people eat different food. When it comes to food choice, no solution is universal. Individual preferences contribute to one’s eating plan. Not considering this aspect of individualization might result in individuals abandoning the dietary management tool.

Motivated by the aforementioned issues, we have developed a customized easy-to-follow dietary tool called *EasyNutrition*. *EasyNutrition* applies the concept of *nutrient profiling* in a mobile-based dietary app and aims to focus individuals’ attention toward nutrition and raise their awareness about the nutritional value of the food recipes they choose. The engine behind *EasyNutrition* is a second artifact that we built to rank different food recipes, the *Intelligent Nutrition Engine*. This algorithm considers the 3 major macronutrients (ie, fat, protein, and carbohydrates), 2 micronutrients (ie, sodium and dietary fibers), and the number of calories the consumer needs daily. To present the nutritional value of a particular food recipe in a simple, easy-to-understand fashion, we adopted the notion of the traffic-light diet. The nutritional information is not given as a strict tricolor output. Rather, it is by analogy a color-coded food rating scale of eight values that rates the food recipe based on its nutrition from red (for extremely non-nutritious choices) to green (for optimal nutritious choices).

### Background

Although genetics are an important consideration in health, during the past half-century, our genes have not measurably altered, and yet, we are significantly more overweight, obese, and prone to lifestyle-related diseases. As of 2014, more than one-third (36.5%) of US adults have obesity, according to the National Health and Nutrition Examination Survey data (2011-2014) [3]; 70.7% are overweight [4]; and 29.1 million Americans (9.3% of the population) are diabetics (as of 2012) [5]. The root of the problem of all these conditions is a poor diet. Tackling this problem is by no means easy and requires complex lifestyle changes. However, a healthy diet is a key component of a healthy lifestyle that can prevent the onset of chronic diseases or mitigate their severity [6].

### Dietary Management

Managing diets is essential when it comes to diet-related chronic diseases. For healthy individuals, it is a preventative measure to maintain a healthy weight and facilitate overall well-being. Essentially, there are 4 different approaches to manage diets, summarized in a study by Arens-Volland et al [6]. These approaches encompass dietary recall, food records, self-management, and menu planning. In the first approach, the patient is asked over the phone about the amount of food and drinks typically consumed in a 24-hour period, along with the method of preparation and the brand of the food items. Using food records, patients will have the chance to do the same by writing down this information. In the self-management approach, a personal digital assistant software is used. The patient will set some predefined goals on what quantities of food to consume. In this case, patients are in charge of how to keep their diet under control no matter what kind of food is consumed. The last approach is menu planning where patients will have their meals planned based on previously identified food preferences. As the first 3 approaches are prone to the tediousness of counting and recording multiple times a day, the focus in this study was to utilize the last approach, which is menu planning.

Many studies have been conducted to develop and evaluate computerized dietary management approaches that are based on diet recall and food records [7,8]. These two methods are designed to both keep track of food intake and count calories or carbohydrates. These apps are promising to assist patients to better manage their diet. However, the notion of calorie counting is very tedious and entails many issues from the patients’ perspective.

First, the underlying method, where users have to log their daily food intake, can suffer from the issue of recall. Examples of these food and calorie tracker apps include *MyFitnessPal*, *Lose It!*, and *Calorie Count*, among many others. These apps allow users to log their food daily, define personal weight loss goals, and review and analyze the gathered data against these goals. In this context, the second issue stems from a realistic limitation, which is the food database where users get to pick recipes from. The quality of the food tracking/dairy app is tightly dependent on this underlying database. However, even the largest current food databases are still far from being complete and often contain only country-specific products [9]. The notion of nutrition education or nutrition profiling helps alleviate these issues as it aims to educate patients about the underlying nutrients that constitute a good or bad food choice.

### Nutrition Education

To better manage diets and sustain a healthy lifestyle, one has to be aware and knowledgeable about the nutritional content of the food consumed. Being educated and aware of macronutrition and micronutrition would contribute to one’s overall health. This knowledge leads to a healthy behavior, which, in turn, holds the promise of preventing the onset of chronic diseases or mitigating their severity. Individualized diet education is effective to both understand diet requirements and control body weight and blood sugar levels [10]. This is especially true when such educational material is delivered in an easy-to-follow and a convenient-to-understand manner. However, knowledge-based nutrition education alone does not change dietary behavior. Behavioral nutrition education is more effective in changing dietary behavior. The effectiveness of the behavioral nutrition approaches was investigated by Bader et al [1]. They conducted a pilot study where they investigated one of the most preferred dietary management approaches: menu planning [11]. The study targeted patients with type 2 diabetes. The authors conducted a single-arm clinical trial to evaluate one of the commercial internet-based menu planning tools. They examined pre- to postintervention changes in body weight, blood pressure, and glycemia among overweight patients with type 2 diabetes mellitus (T2DM; n=33). Nutritional recommendations were operationalized into weekly internet-delivered menu plans. The findings indicated that there was 5% weight reduction in 0.18% of the participants. The study highlighted the effectiveness of the behavioral nutrition approaches that are less structured. These approaches include gaining familiarity with general nutrition principles, acquiring general planning frameworks (eg, carbohydrate exchanges, *points*), and, finally, planning tools to use meal replacements or help prepare meals [1].

### Nutrient Profiling

Nutritional profiling aims to rank food based on their nutritional quality. It is driven by the focus on food quality instead of quantity. Individuals who follow high-score food choices would most likely improve their dietary behavior.

Simplicity is key when it comes to presenting nutritional information. It has been suggested that nutritional information on mobile phones should be easy to read and understand. Arsand et al [12] stated that approximate information is better than accurate facts that are difficult to understand but more precise. It was further suggested that the food information on phones for T2DM should not be too fine-grained because too much detailed information may result in user discouragement and little user friendliness.

This approach emphasizes the idea that carbohydrate and calorie intake counting has become less preferred in favor of more generalized nutritional information about the quality of food compositions. As noted, nutrient profiling is defined as the science of ranking foods according to their nutritional composition for reasons related to preventing disease and promoting health, as stated by the World Health Organization. This idea has led to the creation of many nutritional rating systems.

Driven by the idea of nutrition profiling, the traffic-light diet was developed by Epstein et al in the 1970s [13]. In this dietary approach, Epstein et al [13] used a tricolor palette to create an easy-to-follow diet for overweight children. The notion of a traffic-light diet had inspired new research for two decades because of its groundbreaking nature. The traffic-light diet is a structured eating plan that divides food by the color of the traffic signals. Green (go) is for low-calorie food that can be eaten at any time, orange (caution) is for moderate-calorie food that can be eaten occasionally, and red (stop) is for high-calorie food that should be eaten rarely. Since it was launched, pediatricians have widely used the traffic-light diet to encourage healthy eating habits (EH) among their patients.

Many studies have been conducted utilizing the *traffic-light* dietary approach and have shown promising results. The traffic-light diet is used as a part of a comprehensive treatment, and the results show a significant decrease in obesity in preadolescent children [14,15]. Significant changes in eating patterns have been reported when comprehensive obesity treatment has been combined with the traffic-light diet [7,13]. Reductions in *red foods* have been observed after treatment, with significant associations between changes in intake of *red foods* and weight loss [7] or a decrease in the overweight percentage [13].

In this study, we adopted the presentation approach of the traffic-light diet to present the nutrients contained in a food recipe. However, it was not a strict tricolor output. Rather, it was a color-coded food rating scale of eight values as it takes into consideration five different nutrients and not only the caloric count. It scales food based on its nutritional quality from red, for extremely unhealthy choices, to green, for optimal healthy choices, through intermediate colors.

This study aimed to provide a nutrition educational tool to help people with type 2 diabetes learn about the nutritional content of the food they eat and, hence, improve their dietary behavior by choosing healthier, more nutritional recipes.

### Research Approach

**Figure 1:**
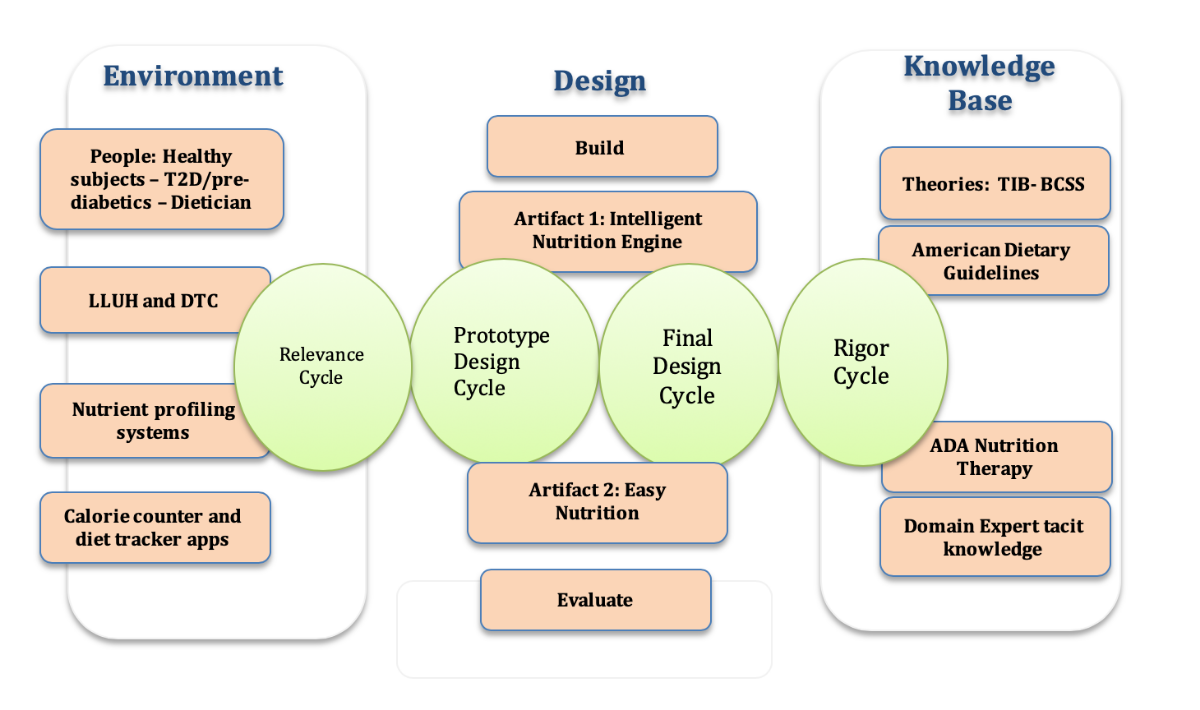
Design science research methodology.

This study followed the design science research (DSR) approach suggested by Hevner and Chatterjee [8]. Hevner et al [16] presented design science as a legitimate research paradigm to be employed in the Information Systems research projects, where the goal was to solve practical problems. DSR aims to build and evaluate technology artifacts in an iterative process. This process can be seen as the embodiment of 3 related cycles of activities: the relevance cycle, the rigor cycle, and the design cycle. The relevance cycle initiates the DSR project by addressing the contextual environment of the research project. The rigor cycle defines the knowledge base of the scientific foundations in the form of theoretical models/theories, experience, and expertise. It aims to ground the DSR artifact to the knowledge base and define its novelty. The design cycle iterates between the essential activities of build and evaluate. The 3 main cycles of DSR are illustrated in Figure 1.

### First Design Artifact: EasyNutrition

**Figure 2:**
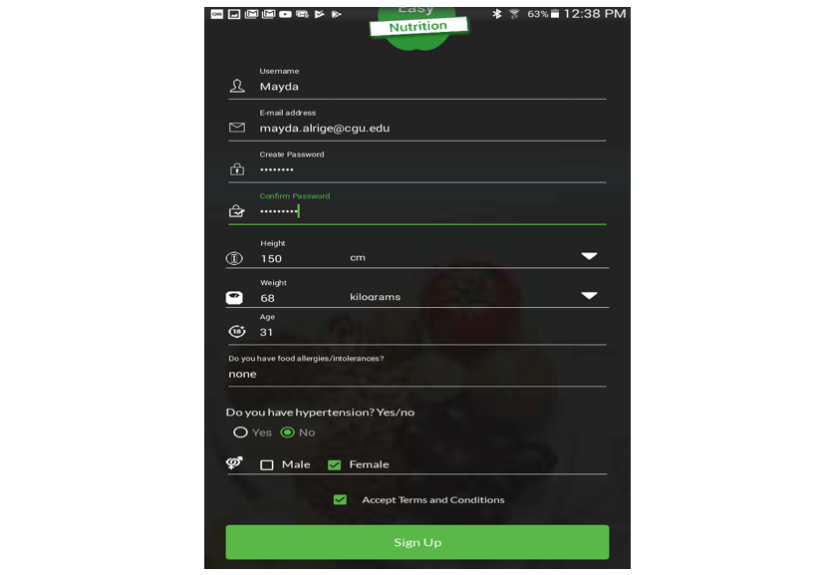
EasyNutrition Sign up screen.

*EasyNutrition* is a customized dietary tool. This means that each user will get cuisine-driven recipes based on his/her own food preference. Once users download the *EasyNutrition* app, they are prompted to sign in or sign up (Figure 2). For new users, *EasyNutrition* will calculate their basal metabolic rate (BMR) based on age, gender, height, and weight. BMR is an estimate of how many calories the user would burn if he/she were to do nothing but rest for 24 hours. In other words, BMR is an estimation of the daily needed calories. In the profile, the user can edit favorite cuisines. These preferences will be saved in the user’s profile and can be easily updated every time the user uses *EasyNutrition*.

**Figure 3:**
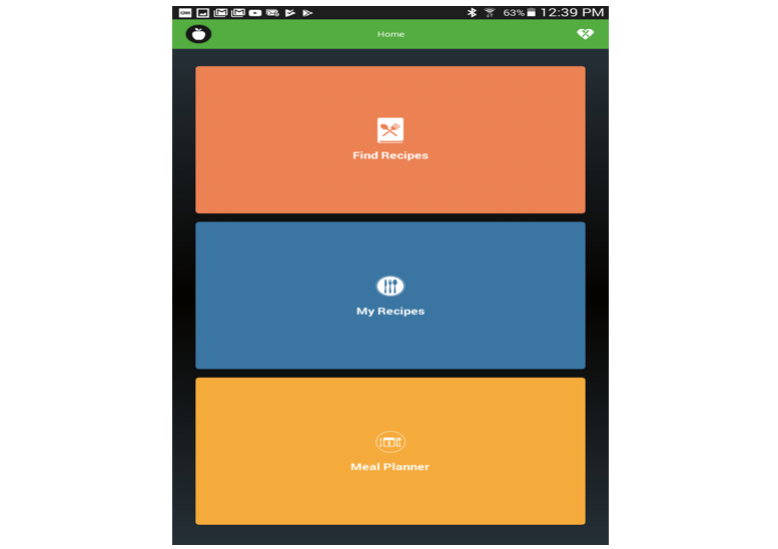
EasyNutrition Home page.

**Figure 4:**
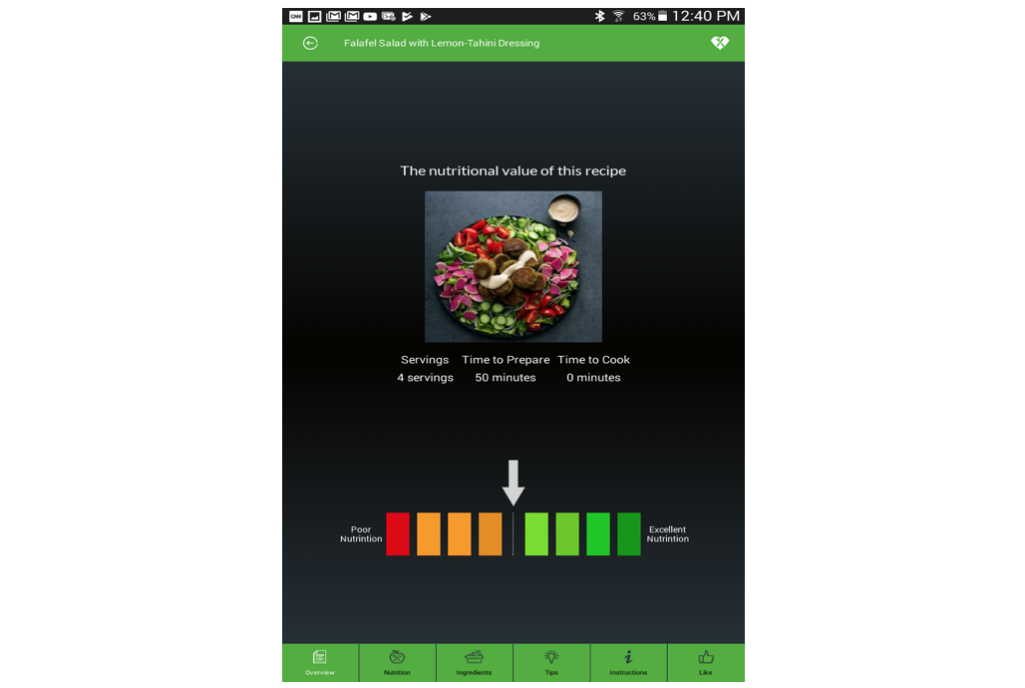
The overall nutritional value of the “Falafel salad” recipe.

Using *EasyNutrition’s* Home page, users can find web-based recipes (tailored to their food preferences as well as their individual nutritional needs), browse the nutritional value of a variety of food recipes, or plan weekly meals. This meal plan can be based on either their favorite recipes (chosen earlier) or their favorite cuisine (Figure 3). In both cases, the resulting recipe will be displayed along with its overall nutritional value. The nutritional value is presented in a traffic-light scale, as can be seen in Figure 4, ranging from red (for a relatively poor nutrition choice) to green (for a relatively excellent nutrition choice) through some intermediate colors.

**Figure 5:**
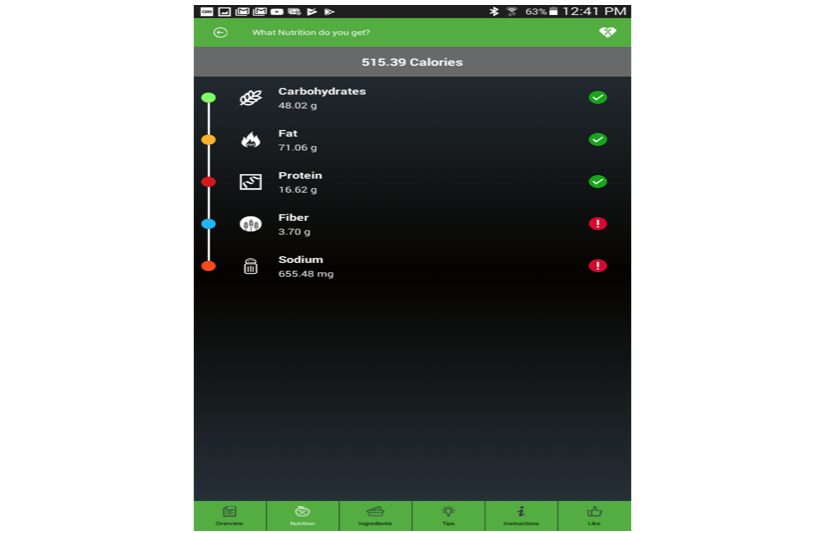
Nutrition break down of the recipe.

This scale will give the user an initial indication of how nutritious the chosen recipe is. If the user is interested to know more about the nutrients that lower the overall nutritional quality, he/she can click on the *nutrition* tab to find out which nutrient is beyond the recommended range, as can be seen in Figure 5. Apart from the nutritional quality, the pertinent information for each recipe is presented. This includes the ingredients, instructions, and some healthy tips on how to maximize the nutritional value of the selected recipe.

As the user saves and adds food recipes to his/her meal plan, he/she will be given either a gold, silver, or bronze trophy based on how active they are with the app. The app will categorize the user into one of the three different groups based on the number of recipes he/she adds to his/her meal plan.

### Second Design Artifact: The Intelligent Nutrition Engine

We developed the *Intelligent Nutrition Engine*, which is a food-ranking algorithm that encompasses 5 different nutrients: the 3 major macronutrients that derive calories (ie, fat, protein, and carbohydrates) and 2 micronutrients (ie, dietary fibers and sodium). The algorithm checks the calories, the percentage of macronutrients, and also the number of micronutrients in a selected recipe. These nutrients determine a healthy or an unhealthy choice. For people with diabetes, we consulted the nutrition therapy recommendations by the ADA [2] to find the important nutrients that have to be considered, along with the appropriate limit for each nutrient. Some of these criteria have slightly different specifications for comorbid type 2 diabetes and hypertension. For example, the sodium recommendation for the general population is less than 2300 mg/day. However, for individuals with both diabetes and hypertension, a further reduction in sodium intake should be individualized (no more than 1500 mg/day). In addition, for comorbid T2DM and hypertension, no more than 7% of fat should come from saturated fat. These criteria were reviewed and validated by the registered dietician from Loma Linda University Medical Center. A list of these nutrients is outlined in Table 1.

### Harris-Benedict Equation

All these nutrients’ amounts/percentages are based on one’s daily calorie intake. As the recommended calorie intake differs based on age, gender, height, and weight, we applied the Harris-Benedict equation to determine the recommended calorie intake or the BMR based on these factors [17]. The final formula encompassing all these nutrients as well as the calories will produce a single holistic number that represents the overall nutritional quality of a particular recipe. The detailed steps of the algorithm can be seen in Textbox 1. For the sake of simplicity, we presented this number in a traffic-light scale that ranges from red to green through some intermediate colors.

**Figure 6:**
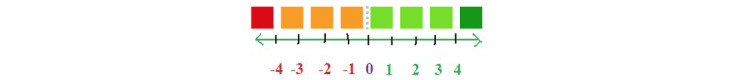
The nutritional score presented behind a traffic-light scale.

The cursor would start right in the middle of the traffic-light scale as an initial score for any given food recipe, as can be seen in Figure 6. The cursor would move to the right if a certain nutrient is within the recommended percentage/amount. The cursor would move to the left if a certain nutrient exceeds the maximum limit or fails to meet the minimum limit of the recommended range. This algorithm was applied to Spoonacular, the largest web-based food Application Programming Interface (API), to rank different food recipes accordingly.

**Table 1:** The nutrition limits per daily recommended percentages/amounts of total calorie intake.

Nutrients		Intake
**Macronutrients**		
	Carbohydrates	45%-65%
	Fats	25%-35% For those with hypertension, no more than 7% of this percentage should come from saturated fat
	Protein	15%-20%
**Micronutrients**		
	Dietary fibers	20-30 grams
	Sodium	No more than 2300 mg No more than 1500 mg daily for diabetics who have hypertension

**The algorithm logic to rank food recipes.**

- Calculate the basal metabolic rate (BMR) based on gender, age, height, and weight based on the Harris-Benedict equation
- Divide the number by 3 assuming 3 meals a day, to get the ideal recipe calories for a particular user
- Recommended recipe calories (RRC)=BMR/3
- Obtain the chosen recipe calories (CRC) and compare the value with RRC

If CRC≤RRC, and is within the recommended range, CRC_w_=1, otherwise CRC_w_=−1

- Obtain the amount of carbs in grams
- Convert this amount into calories (1 gram of carbs provides 4 calories)

0.45% *Rc*≤*C*≤0.65% *Rc*, where *C* is carbs

If it is within the recommended range, C_w_=1, otherwise C_w_=−1; if *C*<0.45%, C_w_=0

- Obtain the amount of fats in grams
- Convert this amount into calories (1 gram of fat provides 9 calories)

0.25% *Rc*≤*F*≤0.35% *Rc*, where *F* is fat

If it is within the recommended range, F_w_=1, otherwise F_w_=−1; if *F*<0.25%, F_w_=0, show the message: *Good job. This choice is low in fat*.

If the patient with diabetes has hypertension, then another test for saturated fat will be conducted: fat sugars should not exceed 0.07% of the total fat

- Obtain the amount of protein in grams
- Convert this amount into calories (1 gram of protein provides 4 calories)

0.15% *Rc*≤*Pr*≤0.20% *Rc*, where *Pr* is protein

If it is within the recommended range, Pr_w_=2, otherwise Pr_w_=−2; if *P*≤15% *R_c_*, P_w_=0

- Obtain the amount of sodium in milligrams

*S*≤450, where *S* is sodium

If *S*≤450, S_w_=1; if *S≥*450, S_w_=−1

- If the patient with diabetes has hypertension, then the amount has a lower cut-off value (<1500/3 mg)
- Obtain the amount of dietary fibers in grams

6 *g*≤*Df*≤10 *g*, where *Df* is dietary fibers

If *Df* is within the recommended range, Df_w_=2; if *Df*>10, Df_w_=3; if *Df*<6, Df_w_=0

## Methods

### Evaluation Plan

**Figure 7:**
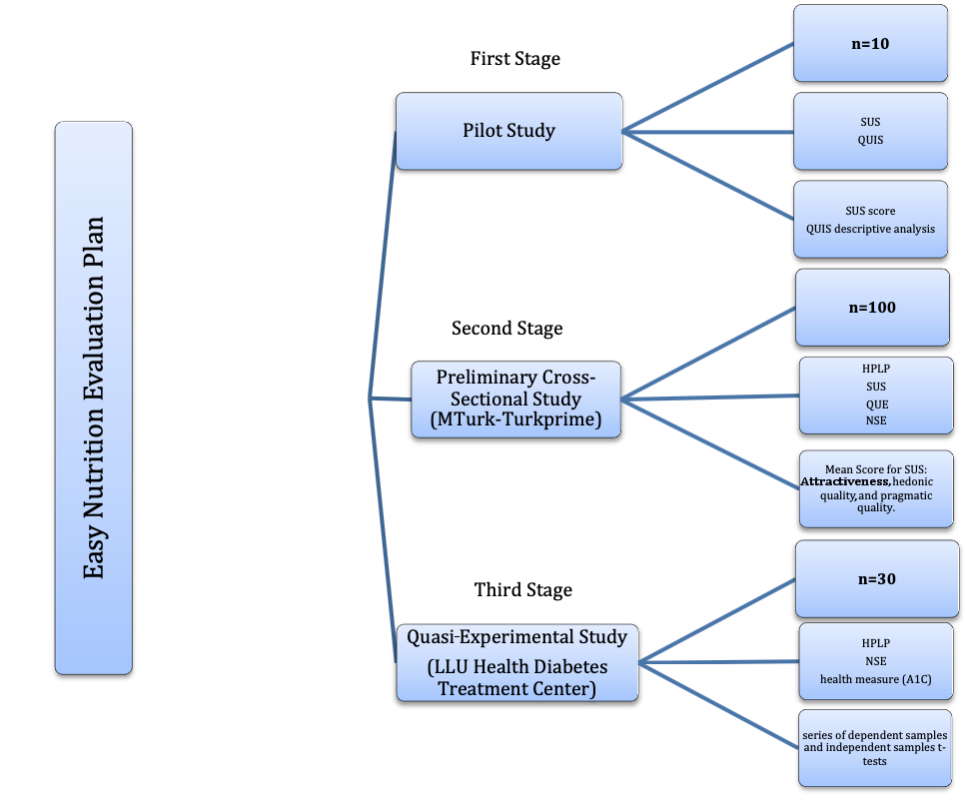
The evaluation plan (subjects, measures, and analysis).

During this study, we evaluated the utility, efficacy, and quality of the app, *EasyNutrition*, from a sociotechnical perspective in three sequential stages (Figure 7). The first stage was a pilot study to test the usability and understandability of *EasyNutrition*’s interfaces. The results can be found in a published paper by Alrige et al [11]. Once we established that the technology is sufficient to use (usable and understandable), we tested the full version of *EasyNutrition* on a wider population in the second stage. The goal was to evaluate *EasyNutrition* for its functionality and quality as a customized dietary tool, and the results were published in a paper by Alrige and Chatterjee [18]. Once we established the usability, quality, and content validity of *EasyNutrition* as a customized dietary tool to help users improve their food choices, we implemented the third stage to test *EasyNutrition*’s efficacy in managing type 2 diabetes. Details about the subjects, study type, measurements, and analysis can be seen in this paper that elaborates on the third stage of the evaluation where we conducted a quasi-experimental field study. In particular, we employed the two-group pretest-posttest design recommended for medical informatics studies to introduce a new intervention [19]. A pre-post intervention study allowed us to capture any change in participants’ dietary behavior/attitude before and after experimenting with *Easy Nutrition.* Both Claremont Graduate University (Institutional Review Board [IRB] number 2964) and Loma Linda University (IRB number 5170397) provided an IRB approval, as we conducted subject recruitment in one of Loma Linda University’s health facilities.

### Setting and Sampling

Subjects were recruited from the diabetes treatment center (DTC), Loma Linda University Health. The center offers classes on diabetes education in 4 different sessions: the first and second sessions are on nutrition, the third one is on physical activity, and the last one is after 2 to 3 months of follow-up with the patients to provide a plan for them to move forward and live with diabetes. Diabetes educators and registered dieticians facilitate those sessions. We attended the sessions to introduce *EasyNutrition* and recruit patients after obtaining their consent. We attended seven classes in the DTC for recruitment, starting from December 20, 2017, and ending on January 10, 2018. The classes were offered during the morning (9 AM-11 AM), afternoon (1:30 PM-3:30 PM), and evening (5:30 PM-7:30 PM) sessions. We followed up with these cohorts of patients during the study course of 3 months before their fourth and last class at the end of their DTC session. The patients were in five different cohorts. The total number of participants was 28, with 5 patients with prediabetes and 23 patients with type 2 diabetes. In total, 12 patients were signed up for the intervention group (group number 1), and 16 patients were signed up for the control group (group number 2). The sample size (n=28) was determined for a two-group, pretest, posttest intervention based on the following test parameters: statistical power=0.80, (medium) effect size=0.5, and significance level=.05.

### Procedures and Measures

In the first class, we started the recruitment by introducing ourselves and our study and explained how relevant it is to their diabetes education session. This included playing a 2-min short demo about *EasyNutrition*. Upon their agreement to participate, subjects completed the following activities.

First, as part of the DTC standard of care (SOC), subjects’ blood was drawn to measure their blood sugar (glycated hemoglobin, HbA_1c_), and this was self-reported to the research personnel. The HbA_1c_ is measured in units of mmol/mol. The HbA_1c_ test measures how much hemoglobin in the blood has become glycated (chemically bonded with glucose).

Second, researchers assigned participants either to the intervention group (group number 1) or to the control group (group number 2), based on the kind of smartphone he/she had (patients with an Android-based smartphone were assigned to group number 1).

Third, participants answered a quick questionnaire about their EH using the Health-Promoting Lifestyle Profile (HPLP) nutrition subscale. The administered questionnaire was adapted from the HPLP, a 48-item questionnaire of self-reported health-promoting lifestyle habits. HPLP consists of 6 scales: self-actualization, health-responsibility, exercise, nutrition, interpersonal support, and stress management. The final structure of the 48-item HPLP was found to have high internal consistency with an alpha coefficient of .922. Sets of items assigned to each of the 6 factors were examined for their reliability as subscales. The nutrition subscale is found reliable with an alpha coefficient equal to .757.

We utilized the Nutrition subscale, a 10-item, 4-point Likert scale that ranges from Never (1), Sometimes (2), Often (3), to Routinely (4) to indicate how often an individual engages in each behavior. The minimum score was 10, and the maximum score was 40 [20].

On the basis of participants’ answers to these questions, we generated a score reflecting their EH. This score was used as a baseline for postcomparison for both groups. An increase in this score suggests improvement in the patient’s dietary behavior. This can establish a potential association between the intervention (interacting with *EasyNutrition*) and health outcomes.

### Intervention

If the patient was assigned to the intervention group, he/she was asked to download the app, *EasyNutrition*, on his/her Android-based phone or tablet. We trained the patients to use the app. Patients were shown how to sign up by entering their basic demographic information (age, height, weight, and gender). They were introduced to the concept of BMR, which is the daily calories their bodies need. They were told that all the nutritional value of the recipes would be based on this BMR value. This is the first form of customization. The second and more important form of customization has been presented by suggesting recipes tailored to the individuals’ favorite cuisines. They were shown how to find web-based recipes and learn about their nutrition breakdown and the quality of ingredients. They were taught how to add the recipes to their meal plan and how to track their usage in their profiles. Every 2 weeks, they received either a text message or a phone call to ask questions about their app usage. Over 3 months, patients in the intervention group used *EasyNutrition* every other week to learn about the nutrition breakdown as well as the nutritional value of a variety of food recipes tailored to their food preferences. Patients in the control group continued receiving the SOC treatment offered by the DTC.

Fourth, at the end of the study, all subjects filled in the same questionnaire about their EH. In addition, their HbA_1c_ was measured again to compare it with the benchmark data from the presurvey data. The follow-up classes at the DTC we attended were at the following dates/times: Monday, February 26, 2018 (5:30-7:30 PM); Wednesday, February 28, 2018 (9 AM-11 AM); Monday, March 26, 2018 (1:30 PM-3:30 PM); and Wednesday, March 28, 2018 (5:30 PM-7:30 PM). One class was designated for only those with prediabetes, and it was offered under the diabetes prevention program. This was on Wednesday, March 14, 2018 (5:30 PM-7:30 PM).

In addition, all subjects were asked to fill in the nutrition self-efficacy (NSE) questionnaire [21]. On the basis of the health belief model [21], the self-efficacy construct has an effect on engaging in health-promoting behavior [22]. Self-efficacy is defined as one’s ability or confidence in making a behavior change. The measurement of patients’ NSE was considered to account for habitual unhealthy behaviors, such as overeating, and test any confounding factors that might have affected their postmeasurement data.

### Analysis

We compared the mean of both eating habit composite scores and HbA_1c_ before and after the study for both groups (intervention and control). Thus, a series of independent and dependent sample one-tailed *t* tests were conducted to test if there was any statistically significant difference within and between groups in terms of patients’ HbA_1c_ and dietary habits. A positive change indicates causality between the intervention and dietary behavior. This would provide preliminary evidence that a potential behavior change might have occurred because of the intervention.

## Results

### Participants Characteristics

Subjects are patients with either type 2 diabetes or prediabetes. Out of the 28 patients who signed up in the third stage, 21 (75%) completed their participation. Of these, 9 patients were men and 12 were women, with the majority being white (11 out of 21). To measure *EasyNutrition* efficacy, we evaluated the subjects’ EH as well as their HbA_1c_ before and after the experiment for both groups.

### Pre-Exposure Analysis

Participants in both groups (9 subjects in the intervention group and 12 subjects in the control group) were administered the presurvey to determine the pre-exposure composite scores of their EH and, hence, establish a benchmark healthy score. The mean composite score for EH was 23.33 for the intervention group and 25.83 for the control group. As for the HbA_1c_, the mean was 8.13 for the intervention group and 7.13 for the control group (Table 2). To ensure that the means of the 2 groups were not significantly different at the start, an independent samples *t* test was conducted on both the composite EH score (t_21_=1.27; *P*=.22) and HbA_1c_ (t_21_=−0.967; *P*=.31). The results in Table 3 show that there is no statistically significant difference between the 2 groups. This illustrates that, before the beginning of the intervention, the difference of the mean between the 2 groups was indeed not significantly different.

**Table 2:** Descriptive statistics on the eating habits composite score and glycated hemoglobin before and after the experiment.

Group			Values	
			Mean (SD)	SE mean
**Intervention (n=9)**				
	**HbA_1c_^a^**			
		Pre-HbA_1c_	8.13 (2.83)	0.94
		Post-HbA_1c_	6.72 (1.27)	0.42
	**EH^b^**			
		Pre-EH	23.33 (3.08)	1.02
		Post-EH	24.33 (3.54)	1.18
**Control (n=12)**				
	**HbA_1c_**			
		Pre-HbA_1c_	7.13 (1.49)	0.43
		Post-HbA_1c_	6.90 (1.64)	0.47
	**Pair 2**			
		Pre-EH	25.83 (5.27)	1.52
		Post-EH	26.08 (2.94)	0.85

^a^HbA_1c_: glycated hemoglobin.

^b^EH: eating habits.

**Table 3:** Pre-exposure independent *t* test.

Group	Levene test		*t* test for equality of means			
	*F* (df=20)	Significance (*P* value)	*t* test (df=19)	Significance (two-tailed; *P* value)	Mean difference	SE difference
Eating habits	3.39	.08	1.27	.22	2.50	1.98
Glycated hemoglobin	6.39	.02	−1.05	.31	−1.00	0.95

### Postexposure Analysis

Both groups exhibited an increase in their composite dietary habits score in the postexposure results and a decrease in HbA_1c_ (Table 4). To objectively measure the changes in the means of the 2 groups, an assessment of statistical significance was conducted through a series of dependent and independent samples *t* tests.

First, a paired samples *t* test was conducted to assess if the changes within the groups are considered statistically significant. The first analysis consisted of two dependent *t* tests that were conducted for each group, individually. The dependent variables are HbA_1c_ and the composite score of EH. As for HbA_1c_ in the control group (n=12), the HbA_1c_ mean dropped from 7.13 to 6.90. This change in the HbA_1c_ was very minimal and not statistically significant (t_12_=1.30; *P*=.22). The intervention group (n=9), however, exhibited a statistically significant change between the pre- and postexposure results of their HbA_1c_ (*t*_9_=2.42; *P*=.04). Their HbA_1c_ had dropped from 8.13 to 6.72 (Table 5).

In addition, the second analysis consists of two independent samples *t* tests to assess the change between the 2 groups after the experiment in terms of both their EH composite score (EH) and HbA_1c_. The results of the independent samples *t* tests showed a statistically significant difference in the postresults between the control and intervention groups in terms of their HbA_1c_ (t_21_=1.94; *P*=.04). However, the difference between the 2 groups in terms of their EH was not statistically significant (t_21_=−.456; *P*=.65; Table 6).

**Table 4:** Changes in both eating habits composite score and glycated hemoglobin.

Group		Before	After
**Intervention**			
	HbA_1c_^a^	8.10	6.60
	EH^b^	23.3	24.4
**Control**			
	HbA_1c_	7.10	6.90
	EH	25.9	26.10

^a^HbA_1c_: glycated hemoglobin.

^b^EH: eating habits.

**Table 5:** Dependent samples *t* test on both glycated hemoglobin and eating habits for both groups.

Group		Values			
		Mean (SD)	SE mean	*t* test (df)	Significance (*P* value)
**Intervention group (n=9)**					
	Pre-HbA_1c_^a^ to post-HbA_1c_	1.41 (1.75)	0.58	2.42 (8)	.04
	Pre-EH^b^ to post-EH	−1.00 (2.74)	0.91	−1.09 (8)	.31
**Control group (n=12)**					
	Pre-HbA_1c_ to post-HbA_1c_	0.23167 (0.62)	0.18	1.30 (11)	.22
	Pre-EH to post-EH	−0.25000 (4.31)	1.24	−0.20 (11)	.84

^a^HbA_1c_: glycated hemoglobin.

^b^EH: eating habits.

**Table 6:** Between-group differences for both glycated hemoglobin and eating habits.

Group	Levene test		*t* test for equality of means			
	*F* (df=20)	Significance (*P* value)	*t* test (df=19)	Significance (two-tailed; *P* value)	Mean difference	SE difference
Glycated hemoglobin difference	12.67	.002	1.94 (19)	.04	1.18	0.54
Eating habits difference	3.30	.09	−0.456 (19)	.65	−0.75	1.64

## Discussion

### Practical Implications

The quasi-experiment conducted for this study showed that there is a statistically significant difference within and between subjects in terms of their HbA_1c_. This provides strong preliminary evidence about the efficacy of using a nutrient profiling–based dietary app to present the nutritional value of different food recipes in a customized meal plan. Combined with the nutrition classes offered by DTC, interacting with *EasyNutrition* was effective in lowering HbA_1c_ in patients with diabetes by focusing their attention on nutrition. This is in line with the findings of Bader et al [1] that customized menu planning is one of the most effective methods for dietary management compared with other methods. The customized menu plans provided by *EasyNutrition* in this study enable patients with type 2 diabetes to learn about nutrition and nutritional value based on what they normally eat.

One might argue that the nutrition classes offered by DTC do have an effect on patients in lowering their HbA_1c_. However, we tried to counterbalance this potential effect by having a control group. Obtaining pretest measurements on both the intervention and control groups allows us to assess the initial comparability of the groups. The assumption is that if the intervention and the control groups are similar at pretest, there is a smaller likelihood of important confounding variables differing between the 2 groups [19]. In addition, we measured the NSE for both groups to account for any habitual unhealthy behavior. The mean of the NSE was 15.25 for the control group and 14.67 for the intervention group (Table 7). The difference was negligible (t_21_=0.49; *P*=.63 as can be seen in Table 8) and indicated that there is a little chance that NSE would have been a confounding factor in this study.

**Table 7:** Nutrition self-efficacy descriptive statistics.

Type		Values	
		Mean (SD)	SE mean
**Nutrition self-efficacy**			
	Control (n=12)	15.25 (3.22)	0.93
	Intervention (n=9)	14.67 (1.66)	0.55

**Table 8:** Nutritional self-efficacy between-group difference.

Type	Levene test		*t* test for equality of means				95% CI of the difference
	*F* (df=20)	Significance (*P* value)	*t* test (df=19)	Significance (two-tailed; *P* value)	Mean difference	SE difference	
Nutritional self-efficacy	2.06	.17	0.49	.63	0.58	1.18	−1.89 to 3.06

For patients with diabetes attending the DTC or any other diet management center, keeping up with a healthy nutrient-dense diet has always been a challenge. According to personnel from the DTC, most patients fall back into their old dietary habits as soon as they leave the center. By having a customized dietary app, such as *EasyNutrition,* in hand, the chances are greater that they will stay motivated enough to adhere to a healthy regimen. This is primarily true especially if the dietary app is presenting the nutrition information in a simple manner that does not interfere with day-to-day activities. Nutrient profiling as utilized in *EasyNutrition* has shown to be beneficial in this regard.

### Research Contribution

This study contributes to the body of knowledge on 2 broad levels: to society and to science. The first level of contribution is a new method for nutrition education. The *Intelligent Nutrition Engine* was developed considering 5 major nutrients (3 major macronutrients and 2 micronutrients) to underpin the nutrient-profiling food rating scale. The *Intelligent Nutrition Engine* classifies different food recipes based on their nutritional value in the form of a traffic-light scale. In fact, nutrition education accounts for a large proportion of diabetes management or the management of any other diet-related chronic disease [6]. Thus, it is important to find a simple, intuitive, easy-to-understand mechanism to present nutritional values. The algorithm developed in this study is based on the notion of behavioral nutrition approaches stressed by Bader et al [1]. The notion behind behavioral nutrition approaches is to increase the likelihood that participants will implement the strategy learned. *EasyNutrition* does not offer general nutritional information. Rather, the nutritional value of the food recipe is presented in a simple, easy-to-understand manner. This study adds to the evidence base that a nutritional behavior strategy may be a modern adjunct to diabetes dietary management. In conjunction with reliable dietary education provided by a registered dietician, the app may have some beneficial effects to improve the dietary behavior of patients with diabetes.

In addition, the steps of the algorithm can be viewed as a set of design principles. The algorithm can be tailored to tackle different health conditions. Both the nutrients and the criteria for each nutrient can be tailored according to the health condition being treated. For example, cardiovascular diseases (CVDs) have certain nutrition therapy recommendations that are slightly different than those for patients with diabetes. Diabetes gives priority to carbohydrate consumption, whereas CVDs give special attention to fat consumption.

The artifact, *EasyNutrition*, signals the second level of contribution. Using DSR, *EasyNutrition* was rigorously developed as a customized, nutrient-profiling tool. *EasyNutrition* applies the Intelligent Nutrition Engine to present the nutritional value of different food recipes in a simple manner. This nutritional value is better absorbed if it is tailored to individual preferences. According to Triandis [17] in his interpersonal behavior theory, habits do influence behavior. Thus, including individual habitual preferences would increase the chance to change dietary behavior toward a healthier one. The design process of *EasyNutrition* was delineated thoroughly through a new DSR framework. This includes design requirement extraction and design feature determination in light of the main design principles followed: customization and simplicity.

### Limitations and Future Work

Although the results of this study are promising, there are some limitations that allow room for further improvement and map out new directions for future research. The first set of limitations concerns the design artifact, *EasyNutrition*, whereas the second set of limitations concerns the Intelligent Nutrition Engine. First, regarding the design of *EasyNutrition*, the app presents the overall nutritional value in a traffic-light scale to promote simplicity. For people with color blindness, this presents an issue as they cannot see the differences of the gradients of the three main colors of the traffic light. Another presentation mechanism can be added above or below the traffic light to mitigate this issue. A simple presentation mechanism such as numerical values or emoticon representations can be utilized. Second, the nutritional value of every recipe does not allow ingredient substitution, that is, if the user ended up cooking one recipe but chose to substitute, for example, white rice with brown rice or decided to grill the chicken breast instead of frying it, the nutritional value is still the same. Dynamic nutritional value calculation would allow users to substitute ingredients and then adjust the overall nutritional value accordingly.

The second set of limitations concerns the Intelligent Nutrition Engine artifact. This algorithm is inspired by the novel concept of *NuVal*, a system that has its own dataset and covers almost all the food products/packaged meals. Owing to financial limitations, we were not able to consult the NuVal dataset or API and utilize the NuVal scores. A good research direction would be to utilize NuVal scores that consider about 30 nutrients and apply them to rank different food recipes. This would give a more comprehensive dietary tool that can possibly present the nutritional value not only for food recipes but also for food products, restaurant meals, and many others.

The third set of limitation concerns the study design. In the third stage of this study, we conducted a quasi-experiment to evaluate the effect of using *EasyNutrition* on managing type 2 diabetes. The sample size was borderline small for the statistics used (n=28). To generalize the results for the effect of using *EasyNutrition* on reducing HbA_1c_ in patients with type 2 diabetes, further research has to be conducted with a larger sample size.

### Conclusions

*EasyNutrition* was developed to introduce and evaluate the novel concept of nutrient profiling in the domain of dietary management apps and investigate its effect on the EH of people with diabetes by focusing their attention toward nutrition and, hence, helping them make better, healthier food choices. In addition, it was developed with cultural differences in mind, that is, all food recipes suggested by *EasyNutrition* are tailored to users’ food preferences. Using *EasyNutrition*, the user can find web-based food recipes tailored to his/her food preferences; learn about their nutrition in a simple, easy-to-understand manner; and plan their weekly meals. *EasyNutrition* has been evaluated in a rigorous 3-stage step-up plan.

In the third stage, participants in the intervention group exhibited significant changes in their HbA_1c_. Participants in the control group, however, exhibited minimal and nonstatistically significant changes in their HbA_1c_. In addition, postresults of the independent samples *t* test showed a statistically significant difference between the control and intervention groups in terms of their HbA_1c_. In conclusion, the results from this design research study illustrate the potential efficacy of using a customized nutrient profiling–inspired dietary app to focus individuals’ attention toward nutrition and improve their EH.

## Abbreviations

ADA: American Diabetes Association
API: Application Programming Interface
BMR: basal metabolic rate
CVD: cardiovascular disease
DSR: design science research
DTC: diabetes treatment center
EH: eating habits
HbA_1c_: glycated hemoglobin
HPLP: Health-Promoting Lifestyle Profile
IRB: Institutional Review Board
NSE: nutrition self-efficacy
SOC: standard of care
T2DM: type 2 diabetes mellitus
